# *Ehrlichia chaffeensis* TRP120 Activates Canonical Notch Signaling To Downregulate TLR2/4 Expression and Promote Intracellular Survival

**DOI:** 10.1128/mBio.00672-16

**Published:** 2016-07-05

**Authors:** Taslima T. Lina, Paige S. Dunphy, Tian Luo, Jere W. McBride

**Affiliations:** a Department of Pathology, University of Texas Medical Branch, Galveston, Texas, USA; b Department of Microbiology and Immunology, University of Texas Medical Branch, Galveston, Texas, USA; c Center for Biodefense and Emerging Infectious Diseases, University of Texas Medical Branch, Galveston, Texas, USA; d Sealy Center for Vaccine Development, University of Texas Medical Branch, Galveston, Texas, USA; e Institute for Human Infections and Immunity, University of Texas Medical Branch, Galveston, Texas, USA

## Abstract

*Ehrlichia chaffeensis* preferentially targets mononuclear phagocytes and survives through a strategy of subverting innate immune defenses, but the mechanisms are unknown. We have shown *E. chaffeensis* type 1 secreted tandem repeat protein (TRP) effectors are involved in diverse molecular pathogen-host interactions, such as the TRP120 interaction with the Notch receptor-cleaving metalloprotease ADAM17. In the present study, we demonstrate *E. chaffeensis*, via the TRP120 effector, activates the canonical Notch signaling pathway to promote intracellular survival. We found that nuclear translocation of the transcriptionally active Notch intracellular domain (NICD) occurs in response to *E. chaffeensis* or recombinant TRP120, resulting in upregulation of Notch signaling pathway components and target genes *notch1*, *adam17*, *hes*, and *hey*. Significant differences in canonical Notch signaling gene expression levels (>40%) were observed during early and late stages of infection, indicating activation of the Notch pathway. We linked Notch pathway activation specifically to the TRP120 effector, which directly interacts with the Notch metalloprotease ADAM17. Using pharmacological inhibitors and small interfering RNAs (siRNAs) against γ-secretase enzyme, Notch transcription factor complex, Notch1, and ADAM17, we demonstrated that Notch signaling is required for ehrlichial survival. We studied the downstream effects and found that *E. chaffeensis* TRP120-mediated activation of the Notch pathway causes inhibition of the extracellular signal-regulated kinase 1/2 (ERK1/2) and p38 mitogen-activated protein kinase (MAPK) pathways required for PU.1 and subsequent Toll-like receptor 2/4 (TLR2/4) expression. This investigation reveals a novel mechanism whereby *E. chaffeensis* exploits the Notch pathway to evade the host innate immune response for intracellular survival.

## IMPORTANCE

*E. chaffeensis* is an obligately intracellular bacterium and the etiologic agent of human monocytotropic ehrlichiosis (HME), an emerging life-threatening tick-borne zoonosis. Mechanisms by which *E. chaffeensis* establishes intracellular infection and avoids innate host defenses are not understood, but functionally relevant host-pathogen interactions with type 1 secreted TRP effectors are essential for the ehrlichial cellular reprogramming strategy. This study provides further insight into the molecular strategies used by obligately intracellular pathogens such as *E. chaffeensis*, which have small genomes and a limited number of effector proteins and exploit evolutionarily conserved host cell programs such as Notch signaling to promote infection and intracellular survival.

## INTRODUCTION

*Ehrlichia chaffeensis* is a Gram-negative obligately intracellular bacterium and etiologic agent of human monocytotropic ehrlichiosis (HME), a group 1 NIAID emerging disease and one of the most prevalent life-threatening tick-borne zoonoses in the United States (1, 2). *E. chaffeensis* exhibits tropism for mononuclear phagocytes and has evolved sophisticated molecular mechanisms to exploit the host cell processes in order to evade immune recognition and destruction by mononuclear phagocytes in which it resides. Cellular reprogramming is dependent in part on host-pathogen interactions associated with newly described type 1 secreted (T1S) tandem repeat protein (TRP) effectors (3–5).

*E. chaffeensis* has a small group of well-characterized TRP effectors, including TRP120, TRP47, and TRP32, which are highly immunoreactive and elicit protective antibodies (6). TRP120 is a major immunoreactive protein expressed by dense-core-form ehrlichiae during infection in both arthropod and mammalian cells and is secreted into the intramorular space, where it translocates to the host cytosol and nucleus (3, 7–9). TRP120 is involved in host cell attachment and entry and was recently shown to function as a nucleomodulin, targeting genes associated with transcriptional regulation, apoptosis, and vesicle trafficking (7, 9, 10). Moreover, TRP120 directly interacts with host target proteins involved in transcriptional and translational regulation, posttranslational modification, immune response, intracellular trafficking, cytoskeletal organization, and apoptosis (11). Notably, TRP120 is also known to interact with the receptor and regulatory components of the Notch and Wnt signaling pathways (9, 11). Recently, we reported that *E. chaffeensis* activates canonical and noncanonical Wnt signaling to facilitate host cell entry and exploits Wnt signaling to promote intracellular survival (10).

The Notch signaling pathway is evolutionarily conserved in eukaryotes and plays important roles in cell proliferation, differentiation, and apoptosis, thereby influencing cell fate (12–15). Three proteolytic cleavage steps are essential for the production of fully functional Notch receptor signaling. The first occurs at site 1 (S1) by furin in the *trans*-Golgi (16, 17), resulting in translocation of the heterodimer to the cell surface. The canonical Notch pathway is activated when the extracellular domain of Notch receptor (NECD) binds to the ligand (DLL1, -3, and -4 and Jagged 1 and -2) expressed on the membrane of neighboring cells. This receptor-ligand interaction results in the exposure of site 2 (S2) in Notch for cleavage by ADAM metalloproteases (18), resulting in NECD shedding and subsequent cleavage of the intracellular domain (NICD) by γ-secretase enzyme (S3 cleavage) (19). NICD translocates to the nucleus, where it forms a triprotein complex with DNA-binding transcription factor RBPjκ (CSL) and transcriptional coactivator Mastermind (MAM), activating Notch target gene transcription (20, 21). We previously demonstrated that TRP120 interacts with the ADAM17 metalloprotease (11) and also acts as a nucleomodulin, binding target genes associated with the Notch signaling pathway, including *notch1* (9).

The Notch pathway is most often functionally associated with cell development and cancer but was recently recognized as an important regulator of innate and adaptive immune responses. The role of Notch signaling in inflammation, autophagy (22), apoptosis (23), Toll-like receptor (TLR) expression (24), T and B cell development (14), and major histocompatibility complex (MHC) class II expression (25) in different cells, including macrophages, has been reported. A role for Notch signaling during bacterial infection has been reported for *Salmonella enterica* serovar Typhimurium, *Mycobacterium bovis*, *Bacillus anthracis*, and *Clostridium difficile*, in which either the bacteria or the secreted toxins altered the Notch pathway to regulate inflammation in the host cell (26, 27).

TLRs are type I transmembrane proteins, which play critical roles in the innate immune response by sensing a diverse set of microbial ligands known as pathogen-associated microbial patterns (PAMPs). TLR2 and TLR4 detect peptidoglycan (PGN) and lipopolysaccharide (LPS) and are the most well-characterized pattern recognition receptors (PRRs) (28). Interaction of PAMPS with the TLRs causes activation of multiple signaling pathways, including NF-κB, extracellular signal-regulated kinase 1/2 (ERK1/2), Jun N-terminal protein kinase (JNK), and p38 mitogen-activated protein kinase (MAPK), which leads to induction of proinflammatory cytokine production and monocyte maturation (29, 30). TLR2 and TLR4 play protective roles against*. E. chaffeensis* infection (25, 31), and *E. chaffeensis* causes decreased expression of TLR2/4 by inhibiting the ERK1/2 and p38 MAPK pathways followed by downregulation of activity of PU.1, a transcription factor required for the expression of TLR2/4 (32–34). However, a mechanistic understanding of *E. chaffeensis* inhibition of ERK1/2 and p38 MAPK pathways and PU.1 is unknown. The TLR, ERK1/2, and p38 MAPK pathways are tightly regulated by multiple signaling pathways, such as integrin CD11b and immunoreceptor tyrosine-based activation-associated receptors (35, 36). Recently, association of Notch signaling in modulation of ERK1/2 and regulation of TLR4-triggered cytokine production was reported (24).

The present study reveals a novel host-pathogen interaction whereby *E. chaffeensis* exploits the Notch signaling pathway to downregulate innate PRRs. We determined that the Notch signaling is activated by *E. chaffeensis*, and Notch activation is directly induced by the T1S effector TRP120. We further analyzed the underlying survival mechanism and showed that *E. chaffeensis* and TRP120-mediated activation of the Notch pathway causes inhibition of the ERK1/2 and p38 MAPK signaling pathways and expression of transcription factor PU.1, which represses TLR2/4 expression. This investigation is the first to demonstrate pathogen exploitation of Notch signaling to modulate PRR expression and to promote intracellular survival.

## RESULTS

### *E. chaffeensis* activates the canonical Notch pathway during infection.

**FIG 1:**
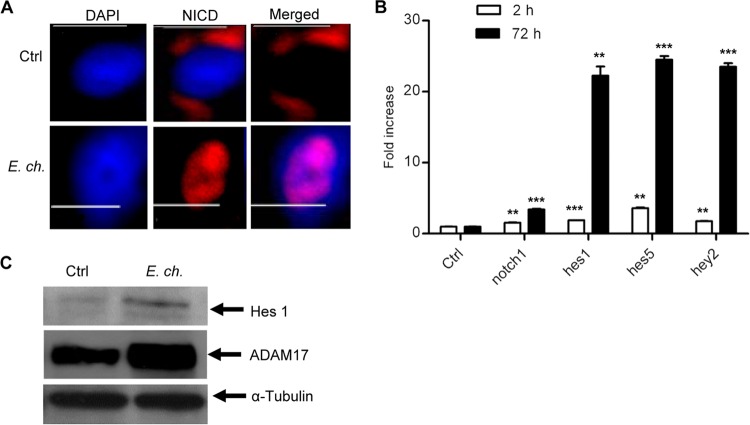
*E. chaffeensis* activates the Notch signaling pathway in THP-1 cells. (A) Nuclear translocation of NICD was analyzed at 2 h p.i. Cells were fixed with paraformaldehyde, permeabilized with Triton X-100, and probed with anti-NICD antibody (Alexa Fluor 568 [red]) and DNA (DAPI [blue]) and then visualized by immunofluorescence microscopy (40×; bars, 10 µm). Ctrl, control; *E. ch.*, *E. chaffeensis*. (B) Expression levels of Notch signaling components in THP-1 cells were analyzed using real-time RT-PCR in uninfected and *E. chaffeensis*-infected THP-1 cells 2 h (open bars) and 72 h p.i. (closed bars). The mRNA levels of *notch1*, *hes1*, *hes5*, and *hey2* were normalized to GAPDH and compared with the level of uninfected cells (Student’s *t* test; **, *P* < 0.01; ***, *P* < 0.001; *n* = 3). (C) Induction of Hes1, ADAM17, and α-tubulin protein expression was analyzed by Western immunoblotting in THP-1 cells at 48 h p.i. Representative data are shown (*n* = 4).

Using Y2H and chromatin immunoprecipitation sequencing (ChIP-seq), we previously reported that *E. chaffeensis* TRP120 interacts with ADAM17 and binds to the promoter region of *notch1* (9, 11). Since *E. chaffeensis* interacts with a component of the Notch signaling receptor complex, we sought to investigate whether *E. chaffeensis* exploits this pathway. Activation of Notch receptor following interaction with its ligand and proteolytic cleavage by the ADAM17 and γ-secretase enzyme involves nuclear translocation of NICD (18, 19). Immunofluorescence microscopy was used to measure NICD expression and localization in uninfected and *E. chaffeensis*-infected cells. NICD translocation to the nucleus was observed within 2 h of *E. chaffeensis* infection (Fig. 1A). Since nuclear translocation of NICD results in activation of specific Notch target genes (20, 21), next the expression of different Notch signaling components and target genes were examined in *E. chaffeensis*-infected cells. The most important and well-characterized Notch target genes are the families of basic helix-loop-helix proteins, hairy and enhancer of split (Hes) and hairy and enhancer of split with YRPW motif (Hey) (37). These DNA binding proteins function as transcriptional repressors and are the primary effectors of Notch signaling. Reverse transcription-PCR (RT-PCR) data showed *notch1*, *hes1*, *hes5*, and *hey2* mRNA expression was significantly increased as early as 2 h postinfection (p.i.), reaching a maximum at 72 h p.i. (Fig. 1B). Consistent with RT-PCR data, increased expression of Hes1 and ADAM17 protein was also observed by Western immunoblotting after 2 days p.i. The housekeeping protein α-tubulin was unchanged (Fig. 1C). Collectively, these results demonstrate that *E. chaffeensis* activates the canonical Notch signaling pathway during infection.

### Analysis of Notch signaling pathway gene expression during *E. chaffeensis* infection.

**FIG 2:**
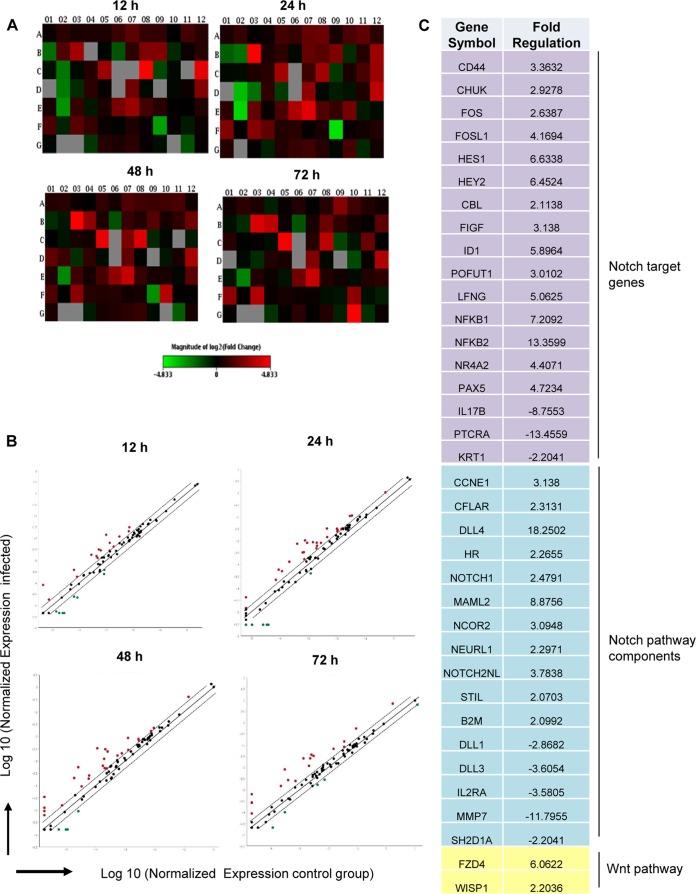
Expression array analysis of Notch signaling pathway genes during *E. chaffeensis* infection. (A) Heat map showing relative expression levels of Notch signaling pathway components and the downstream target genes at 12, 24, 48, and 72 h p.i. Each well in the heat map represents an individual gene, and the scale bar shows color-coded differential expression (where red indicates induction and green indicates repression) from the mean gene expression level of uninfected cells. The degree of color represents the level of induction/repression. (B) Scatter plot showing the Notch PCR array data. A red dot represents upregulation, a black dot represents no significant change of expression, and a green dot represents downregulation. (C) List of genes with their fold change which showed differential expression (up- and downregulation) at 24 h p.i. Purple, blue, and yellow color codes, respectively, have been used for Notch signaling pathway target genes, Notch pathway components, and other genes involved in signaling pathway that cross talk with the Notch signaling pathway.

To understand the global effect of *E. chaffeensis* on the Notch signaling pathway and to examine ehrlichial activation of this pathway, a transcriptional analysis was performed to examine Notch-regulated gene expression. A human Notch signaling PCR array consisting of 84 genes, including Notch binding and receptor processing genes, the putative Notch target genes, genes from Sonic Hedgehog and Wnt receptor signaling pathways that cross talk with the Notch signaling pathway (see Fig. S1 in the supplemental material for gene table) was used. Heat maps were constructed depicting the differential expression of the Notch signaling pathway genes in the *E. chaffeensis*-infected and uninfected cells (Fig. 2A). The intensities of the red and green in the heat map represent the levels of induction and repression, respectively. PCR array data identified activation of canonical Notch signaling pathway by *E. chaffeensis* at 12, 24, 48, and 72 h p.i. The expression patterns of genes that were consistently upregulated throughout all different time points included the Notch target genes *hes1*, *hey2*, *NFκB1*, *NFκB2*, *nr4a2*, *pax5*, *fosl1*, *chuk*, and *ccne1* and Notch pathway component genes—e.g., *notch1* (receptor), *dll4* (ligand), and *maml2* (transcription complex protein). The transcription factor gene *rbpjκ* and E3 ubiquitin ligase gene *dtx1*, which play important roles in Notch pathway activation and regulation, were upregulated at 48 and 72 h p.i. Only a small percentage of genes were downregulated during the infection, including genes for the Notch pathway components *dll1* and *mmp7* (Fig. 2A). In Fig. 2B, the scatter plot shows the comparison of the normalized expression of all genes in the Notch PCR array between infected and uninfected cells. The central line indicates unchanged gene expression (2-fold regulation cutoff), the red dots represent the genes that were upregulated, the green dots represent genes that were downregulated, and the black dots represent genes with no significant difference in expression level. Although the gene expression patterns were similar throughout the different time points, maximum changes in Notch gene expression occurred at 24 h p.i. When the differential expression of these individual genes was analyzed, 38 genes showed significant differential expression (*P* < 0.05), including 28 (33.33%) that were upregulated and 10 (11.90%) that were downregulated (Fig. 2C). These results support canonical Notch signaling pathway activation during early and late phase of *E. chaffeensis* infection.

### The Notch signaling pathway is required for *E. chaffeensis* survival.

**FIG 3:**
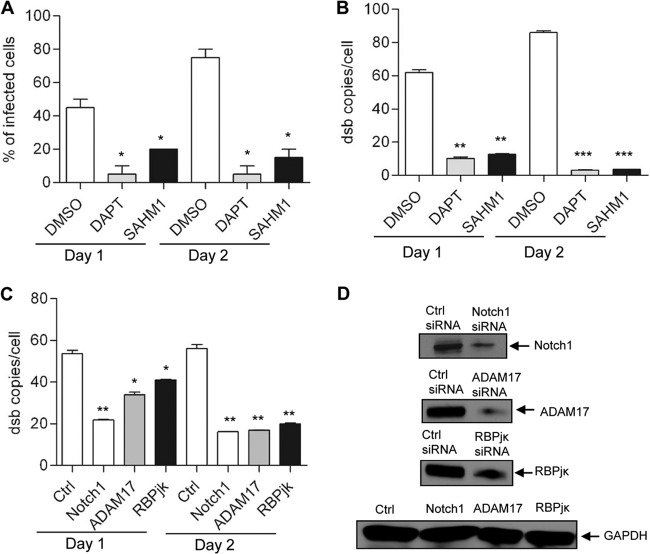
Inhibition of the Notch pathway decreases *E. chaffeensis* load. THP-1 cells were treated with pharmacological inhibitors against γ-secretase enzyme (DAPT) and RBPjκ transcription complex (SAHM1). Cells were infected with *E. chaffeensis* after 1 h posttreatment. Ehrlichial loads were determined at 24 and 48 h p.i. either by (A) calculating the percentage of infected cells by counting 100 Diff-Quik-stained cells or (B) using qPCR measurement of *dsb* copy number. (C) THP-1 cells were transfected with specific or control siRNA to knock down Notch1/ADAM17/RBPjκ and then infected with *E. chaffeensis* (1 day posttransfection). Ehrlichial loads were determined using qPCR measurement of *dsb* copy number at 24 and 48 h p.i. Data are represented as means ± SD (*, *P* < 0.05; **, *P* < 0.01; ***, *P* < 0.001; *n* = 3). (D) Western blots confirmed the reduction of Notch1, ADAM17, and RBPjκ proteins at day 2 p.i.

Notch signaling not only is required for cell growth and proliferation, but also plays important role in determining the fate of mature immune cells (14, 15). Since the Notch pathway regulates both innate and adaptive immune responses, and this pathway is activated during *E. chaffeensis* infection, the role of this pathway in ehrlichial survival was examined. To that end, cells were treated with Notch signaling transcription complex inhibitor SAHM1 and γ-secretase inhibitor DAPT {*N*-[*N*-(3, 5-difluorophenacetyl-l-alanyl)]-*S*-phenylglycine *t*-butyl ester}. SAHM1 is a cell-permeable small peptide that targets critical protein-protein interaction in the Notch transcription complex and prevents their assembly (38). The γ-secretase inhibitor DAPT is a dipeptide and targets the C-terminal fragment of presenilin that is a component of γ-secretase protein (39). THP-1 cells were treated with different concentrations of SAHM1 (1, 5, and 10 µM) and DAPT (0.5, 1, and 5 µg/ml), and cells were infected with cell-free *E. chaffeensis* (at a multiplicity of infection [MOI] of 50). A dose-dependent effect of both of inhibitors on bacterial load was observed as the percentage of ehrlichia-infected cells was determined using Diff-Quik staining (see Fig. S2A to S2D in the supplemental material). A significant decrease in bacterial load in cells treated with 10 µM SAHM1 and 5 µg/ml DAPT was observed; therefore, this concentration was used in additional experiments. There was a >50% decrease in percentage of infected cells after inhibition of the Notch signaling pathway at day 1 p.i. and >80% inhibition at day 2 p.i. (Fig. 3A), and real-time qPCR detected an ~90% decrease in bacterial load in the presence of Notch inhibitors (Fig. 3B). No apparent cell death or toxicity was observed with inhibitor treatment within the experimental window, as cell viability measured by trypan blue (data not shown). To obtain additional evidence supporting the role of Notch pathway in *E. chaffeensis* survival, small interfering RNAs (siRNAs) were used to knock down expression of the receptor Notch1, metalloprotease ADAM17, and transcription factor RBPjκ in THP-1 cells, and then the cells were infected with *E. chaffeensis*. These components were selected since they play critical role in canonical Notch signal transduction. Bacterial load was measured using real-time quantitative PCR (qPCR) by amplification of the integral ehrlichial gene *dsb*. Consistent with Notch pathway inhibitor experiments, significant reduction of bacterial load was also found in cells in which Notch component genes were knocked down (Fig. 3C). Protein expression of Notch1, ADAM17, and RBPjκ was reduced in siRNA-transfected cells compared with that in the control siRNA-transfected cells (Fig. 3D). Overall, the results obtained from pharmacological inhibitors and siRNA experiments support the importance of Notch signaling in *E. chaffeensis* survival.

### *E. chaffeensis* TRP120 protein interacts with Notch receptor complex.

**FIG 4:**
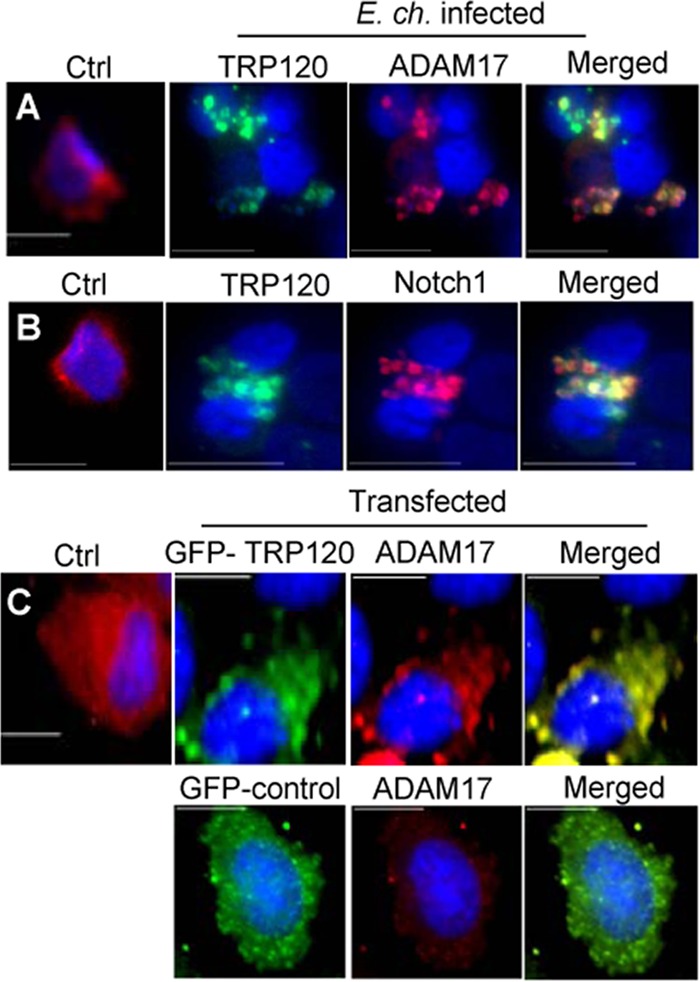
ADAM17 and Notch1 interact with TRP120-expressing *E. chaffeensis*. *E. chaffeensis*-infected or uninfected THP-1 cells (48 h) were fixed, permeabilized, and probed with (A) anti-TRP120 (green) and anti-ADAM17 (red) or (B) anti-TRP120 (green) and anti-Notch1 (red). ADAM17 and Notch1 protein colocalization with TRP120 was observed. (C) HeLa cells were transfected with GFP-TRP120 wild-type (WT) or GFP control plasmids and probed with anti-ADAM17 (red) antibody (24 h posttransfection). Direct ADAM17 and TRP120 interaction through colocalization was observed. Cells were visualized by immunofluorescence microscopy (40×; bars, 10 µm). DAPI shows DNA (blue). Representative data are shown (*n* = 4).

*E. chaffeensis* activates canonical Notch signaling, which is requisite for survival. However, the mechanism of *Ehrlichia*-induced Notch activation remains undefined. *E. chaffeensis* TRP effectors are among the major immunoprotective proteins and contain species-specific epitopes (40). TRP120 functions as an adhesin, facilitating ehrlichial entry, and is a nucleomodulin (7). Moreover, our Y2H data showed TRP120 interacts with ADAM17 and binds to the promoter region of *notch1* (9, 11). We hypothesized that TRP120 interaction with the Notch receptor complex components is required for activation of the Notch pathway. In order to further examine the distribution and colocalization of ADAM17 with the TRP120-expressing ehrlichial inclusions, cells were stained with anti-ADAM17 and anti-TRP120 antibody. Immunofluorescence microscopy showed diffused cytoplasmic localization of ADAM17 in uninfected THP-1 cells; however, consistent with previous Y2H data, colocalization of ADAM17 with morulae expressing TRP120 was observed (Fig. 4A). Since Notch1 and ADAM17 are components of the receptor complex, colocalization of Notch1 with *E. chaffeensis* morulae was also examined. In contrast to the diffused cytoplasmic localization of Notch1 in uninfected cells, colocalization of Notch1 with TRP120-expressing morulae was observed (Fig. 4B). These data were further validated by transfecting HeLa cells with green fluorescent protein (GFP)-tagged TRP120 or GFP control plasmids. Colocalization of ADAM17 and TRP120 was observed (Fig. 4C), demonstrating that Notch receptor components are associated with ehrlichial vacuoles and confirms previous TRP120-ADAM17 interaction data.

### TRP120 activates the canonical Notch signaling pathway.

**FIG 5:**
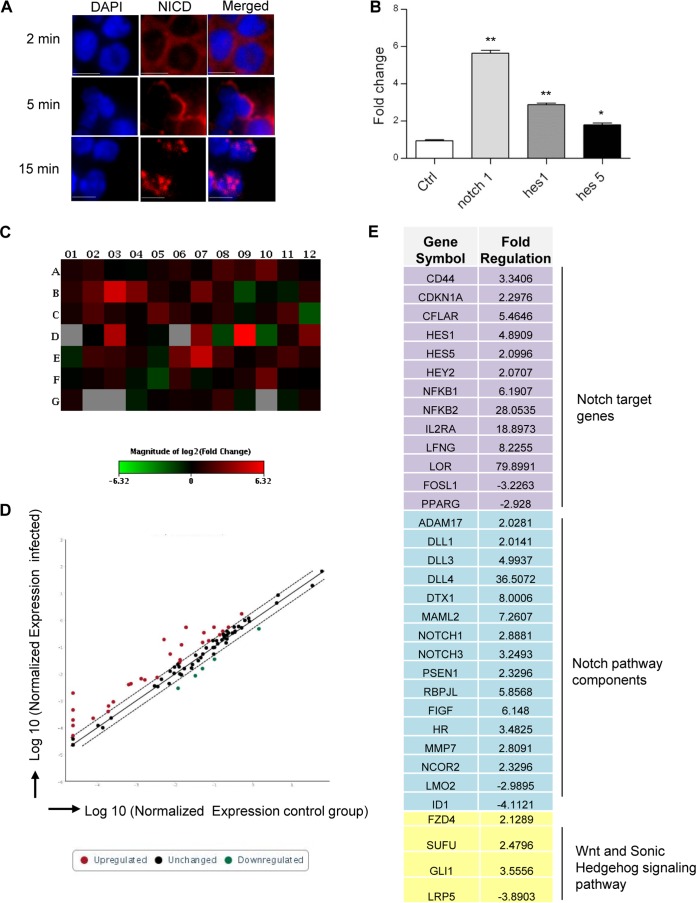
Activation of the Notch signaling pathway by *E. chaffeensis* TRP120. THP-1 cells were treated with TRP120-coated beads, fixed, permeabilized, and probed with anti-NICD (red) and DNA (DAPI [blue]) and then visualized by immunofluorescence microscopy (40×). NICD nuclear translocation after 15 min of stimulation with TRP120-coated beads was observed (bars, 10 µm). (B) Expression levels of *notch1*, *hes1*, and *hes5* in THP-1 cells were analyzed using real-time RT-PCR. RNA was isolated from cells stimulated with TRP120- or thioredoxin-coated beads (2 h). The mRNA level was normalized to GAPDH and compared with the level of control cells (Student’s *t* test; *, *P* < 0.05; **, *P* < 0.01; *n* = 3). The Notch pathway PCR array was performed to analyze the gene expression level in THP-1 cells stimulated with TRP120-coated beads compared to that in the thioredoxin control (24 h). (C) Heat map showing the expression level of Notch signaling genes after TRP120 expression level of thioredoxin-stimulated cells. The degree of color represents the level of induction (red)/repression (green). (D) Scatter plot showing Notch gene expression. A red dot represents increased gene expression, a black dot represents no significant change of expression, and a green dot represents decreased gene expression compared to control cells. The cutoff was 2-fold. (E) List of genes with their fold change which showed differential expression (up- and downregulation) at 24 h poststimulation with TRP120-coated beads.

To further examine the role of TRP120 in Notch pathway activation, TRP120-coated FluoroSpheres (sulfate microsphere beads) were used to stimulate the THP-1 cells. Notch activation involves proteolytic release of NICD from Notch by the furin, ADAM17, and γ-secretase enzymes and translocation of NICD to the nucleus. Thus, the expression of NICD was used to monitor activation of Notch pathway. Since human monocytes constitutively express Notch receptors and ligands at basal levels, we confirmed NICD basal-level expression in the cytoplasm of untreated cells. However, within 5 min of stimulation, condensed expression of NICD was observed near the nucleus, which then translocated to nucleus within 15 min of stimulation (Fig. 5A). Since thioredoxin tag was used as the TRP120 fusion protein, cells were treated with thioredoxin as a control. Nuclear translocation of NICD was not observed in untreated cells or in cells treated with thioredoxin. To further delineate the role of TRP120 modulating Notch pathway gene expression levels, cells were stimulated with TRP120-coated beads for 2 h, and the gene expression levels of *notch1* and the Notch pathway target genes *hes1* and *hes5* were examined. A significant increase in expression of all of the selected genes in TRP120-treated compared to thioredoxin-treated cells was detected (Fig. 5B). To further establish the role of TRP120 in induction of the Notch signaling pathway, we globally analyzed the expression pattern of genes involved in Notch pathway using the human Notch signaling PCR array. The heat map in Fig. 5C shows the graphical representation of gene expression patterns of all 84 genes involved in Notch signaling, in cells stimulated with TRP120 and normalized to control cells treated with thioredoxin. Examination of individual gene expression identified 33 (39.3%) genes were differentially expressed (*P* < 0.05). The scatter plot in Fig. 5D shows differential expression of Notch pathway genes during TRP120 stimulation compared to the thioredoxin control, where the up- and downregulation of genes are represented as red and green dots (2-fold cutoff), respectively. Figure 5E shows the list of genes and the fold change during the TRP120 stimulation. TRP120-stimulated cells showed increased expression of 16 Notch pathway components (19%) including receptors (*notch1* and *notch3*), ligands (*dll1*, -*3*, and -*4*), transcription factor complex proteins (*maml2* and *rbpjκ*), γ-secretase protein (*psen1*), and the TRP120-interacting enzyme (*adam17*). Significant induction of 12 target genes (14%), including *hes1*, *hes5*, *hey2*, *NFκB 1*, *NFκB 2*, *il2ra*, and *lor*, and downregulation of 4 genes (5%) (*lmo2*, *id1*, *fosl1*, and *lrp5*) was also observed. Previously, we demonstrated that ehrlichial TRPs, including TRP120, are secreted during infection (3); therefore, we were also interested in exploring whether soluble TRP120 could potentially modulate Notch signaling in neighboring cells. Approximately 65% of the Notch signaling pathway component and target gene exhibited significant differential expression when stimulated with soluble TRP120 (see Fig. S3A and B in the supplemental material). Together these data demonstrate that TRP120 independently and efficiently activates the canonical Notch signaling pathway. Moreover, these findings also suggest that ehrlichial infection not only manipulates the host Notch signaling pathway through direct interactions between ehrlichiae and host cells but potentially involves uninfected neighboring cells through the release of soluble TRP120 during the exit phase.

### *E. chaffeensis* represses the ERK1/2 and p38 MAPK pathways through Notch signaling.

**FIG 6:**
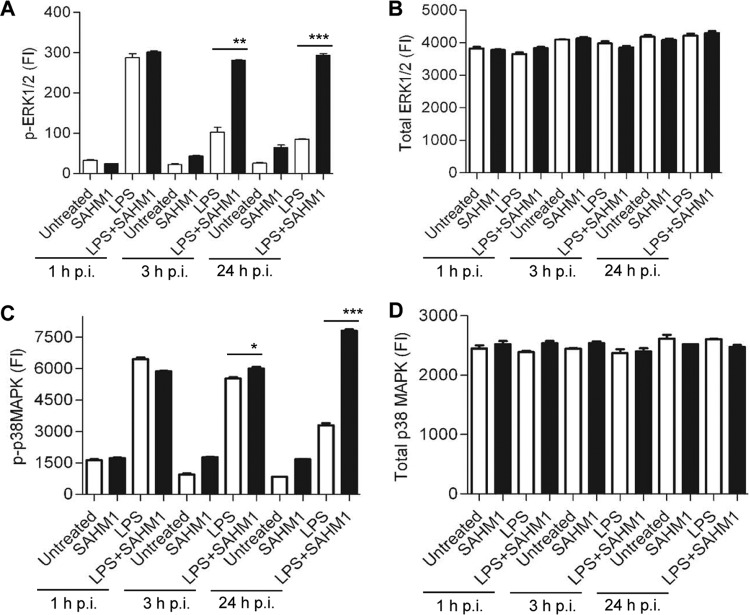
Notch signaling regulates ERK1/2 and p38 MAPK signaling during *E. chaffeensis* infection. THP-1 cells were infected with *E. chaffeensis* (MOI of 100; 1 h posttreatment) in the presence or absence of notch inhibitor SAHM1 (10 µM). Medium or LPS (100 ng/ml) was added at the indicated times, and cells were incubated for 30 min. Cells were lysed after incubation, and cell lysates were analyzed for (A) phosphorylated ERK1/2 (p-ERK1/2), (B) total ERK1/2, (C) phosphorylated p38 MAPK (p-p38MAPK), and (D) total p38 MAPK using Luminex bead arrays. Results represent the mean ± SD (*, *P* < 0.05; **, *P* < 0.01; ***, *P* < 0.001; *n* = 4).

Previous studies reported downregulation of PU.1 and TLR2/4 expression during *E. chaffeensis* infection and demonstrated that host cells become progressively less responsive to LPS-mediated stimulation. Moreover, the underlying mechanism involved inhibition of the ERK1/2 and p38 MAPK pathways (34). Since recent studies linked the Notch signaling pathway with inhibition of TLR-triggered inflammation and inhibition of ERK1/2 (24), we sought to determine the role of *E. chaffeensis*-mediated activation of Notch signaling in inhibition of the ERK1/2 and p38 MAPK pathways. Therefore, the phosphorylated and total levels of ERK1/2 and p38 MAPK protein were examined in response to *E. chaffeensis* infection and LPS stimulation in the presence and absence of Notch transcription factor inhibitor SAHM1. Decreased levels of phosphorylated ERK1/2 in response to LPS were detected within 3 h of ehrlichial infection. In contrast, inhibition of ERK1/2 phosphorylation was blocked in the absence of Notch signaling (Fig. 6A). However, the total level of ERK1/2 remained unchanged (Fig. 6B), and the level of phosphorylated p38 MAPK decreased beginning at 3 h p.i. and was significantly downregulated at 1 dpi, resulting in decreased responsiveness of p38 MAPK to LPS. However, phosopho-p38 MAPK levels were not decreased when the cells were pretreated with SAHM1 (Fig. 6C), and the total level of p38 MAPK remained unchanged (Fig. 6D). These results suggest that activation of Notch signaling plays a key role in downregulation of the ERK1/2 and p38 MAPK pathways during *E. chaffeensis* infection.

### *E. chaffeensis*-mediated PU.1 inhibition and TLR2/4 downregulation depend on the canonical Notch signaling pathway.

**FIG 7:**
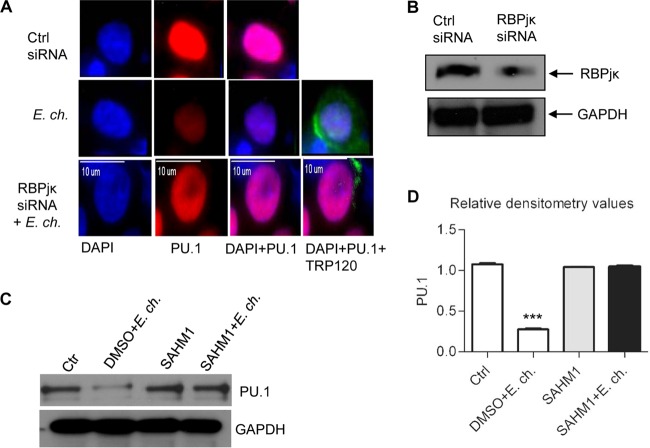
*E. chaffeensis*-mediated downregulation of PU.1 depends on the Notch signaling pathway. THP-1 cells were transfected with either control siRNA or RBPjκ siRNA, incubated for 24 h, and then infected with *E. chaffeensis*. After 24 h p.i., cells were stimulated with LPS (100 ng/ml) for 1 h, and expression of PU.1 was determined (A) by probing with anti-PU.1 (red) and anti-TRP120 (green) and visualized by immunofluorescence microscopy (bars, 10 µm) or by using (B) Western blotting to confirm the reduction of RBPjκ protein. (C) Western blot of whole-cell lysates of uninfected and *E. chaffeensis*-infected cells (48 h) in the presence of vehicle (DMSO) or Notch inhibitor SAHM1. Representative data are shown (*n* = 4). (D) Quantitative analysis of the Western blot data using ImageJ software (Student’s *t* test; ***, *P* < 0.001; *n* = 4).

To investigate the role of Notch signaling in regulation of PU.1 expression, RBPjκ (Notch transcription factor) expression was silenced in THP-1 cells with specific siRNA. Control siRNA and RBPjκ siRNA-transfected cells were infected with *E. chaffeensis* 1 day posttransfection and stimulated with LPS (100 ng/ml) for 1 h after 1 day p.i. Using immunofluorescent microscopy, high levels and predominant localization of PU.1 in the nucleus of uninfected and control siRNA-treated THP-1 cells were observed, but there was a reduction of expression in *E. chaffeensis*-infected cells. However, we observed reconstitution of the PU.1 expression level in the nucleus of THP-1 cells which were treated with RBPjκ siRNA to inhibit Notch signaling before infection (Fig. 7A). Protein expression of RBPjκ was reduced in specific siRNA-transfected cells compared to control siRNA-treated cells (Fig. 7B). To independently confirm the results seen in immunofluorescence microscopy, PU.1 protein levels during *E. chaffeensis* infection in whole-cell lysates in the presence or absence of the Notch transcription factor inhibitor SAHM1 were determined by Western blotting. As shown in Fig. 7C, the level of PU.1 was reduced in *E. chaffeensis*-infected cells compared to that in controls. However, no inhibition was seen in *E. chaffeensis*-infected cells, which were treated with Notch inhibitor SAHM1. Densitometry data generated by the software ImageJ and normalized with the housekeeping protein glyceraldehyde-3-phosphate dehydrogenase (GAPDH) showed significant differences in the level of PU.1 in *E. chaffeensis* infected cells compared to those in control and inhibitor-treated cells (Fig. 7D). Thus, the Western blot and immunofluorescence assay (IFA) data were consistent and indicated that the Notch signaling was responsible for *E. chaffeensis* mediated inhibition of PU.1.

**FIG 8:**
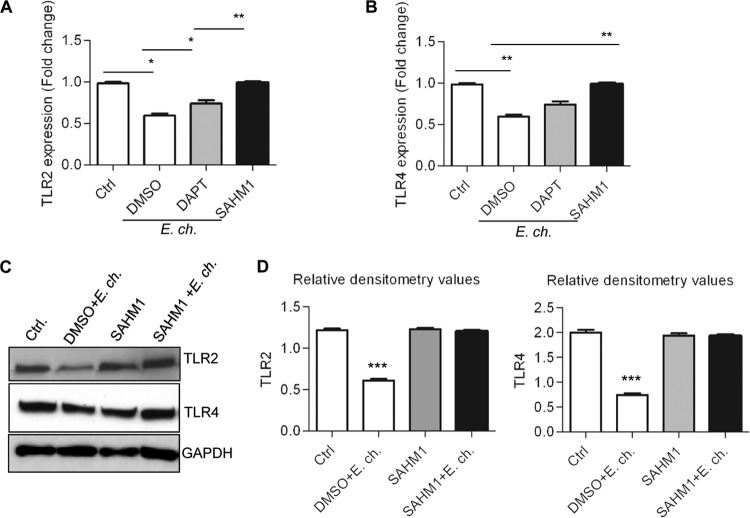
The Notch signaling pathway plays a critical role in inhibition of TLR2/4 expression during *E. chaffeensis* infection. THP-1 cells were treated with either vehicle (DMSO) or Notch inhibitors DAPT and SAHM1 for 1 h and then infected with *E. chaffeensis*. Expression of TLR2 and -4 was measured using RT-PCR (24 h p.i.) and Western blotting (48 h p.i.) after 1 h of stimulation with LPS (100 ng/ml). (A) TLR2 and (B) TLR4 mRNA levels were normalized to GAPDH and compared with the levels in the untreated cells (*, *P* < 0.05; **, *P* < 0.01; *n* = 3). (C) Immunoblot analysis was done using anti-TLR4 and anti-TLR2 antibodies. Representative data are shown (*n* = 4). (D) Relative band intensities of TLR2/4 have been normalized to the loading control GAPDH and were determined using ImageJ software (Student’s *t* test; *, *P* < 0.05; **, *P* < 0.01; ***, *P* < 0.001; *n* = 4).

To demonstrate that the Notch-induced inhibition of PU.1 expression resulted in downregulation of TLR2/4 expression, THP-1 cells were infected with *E. chaffeensis* in the presence or absence of Notch inhibitors (e.g., DAPT and SAHM1). After 24 h p.i., cells were treated with LPS (100 ng/ml) for 1 h. As shown in Fig. 8A and B, *E. chaffeensis* infection caused significant decrease in TLR2 and -4 expression compared to the level in uninfected cells even after LPS stimulation. However, *E. chaffeensis* was unable to downregulate TLR2/4 expression when Notch signaling was blocked. Western blot analysis of THP-1 cells treated under the same conditions provided an independent approach to validate differential expression of these PRRs in the whole-cell lysate of *E. chaffeensis*-infected cells (2 days p.i.) in the presence or absence of Notch signaling. Immunoblot data correlated with gene expression results, showing reduced expression of TLR2/4 proteins in *E. chaffeensis*-infected cells compared to uninfected cells, whereas in the presence of Notch signaling inhibitor, differences in TLR expression compared to the uninfected control were not observed (Fig. 8C). In Fig. 8D, densitometry data show quantitative comparison of the Western blot where the levels of TLR2/4 were normalized with the housekeeping protein GAPDH. Collectively, these data support a key role of Notch signaling in TLR2/4 downregulation during *E. chaffeensis* infection.

### TRP120 effector protein plays a crucial role in inhibition of TLR2/4 response.

**FIG 9:**
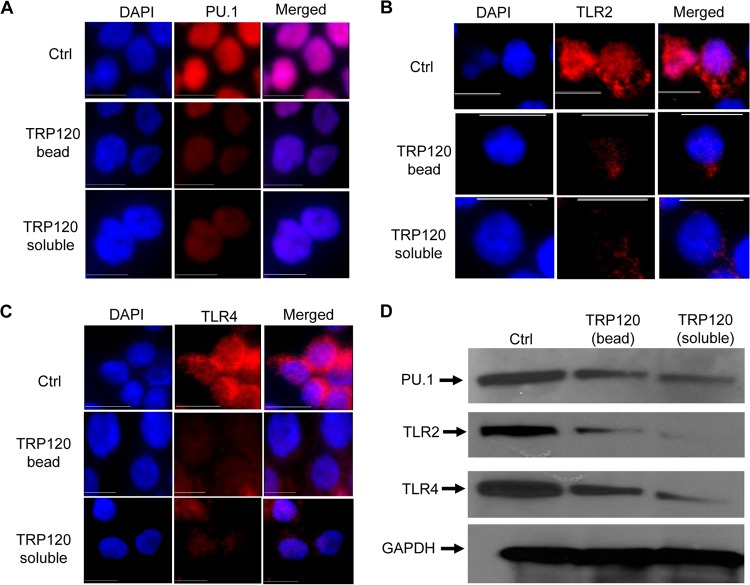
TRP120-mediated downregulation of PU.1, TLR2, and TLR4 expression. THP-1 cells were treated with thioredoxin (control)- or TRP120-coated latex beads or TRP120 in suspension (1 µg/ml) for 24 h and stimulated with LPS for 1 h. IFA analysis was done to measure (A) PU.1, (B) TLR2, and (C) TLR4 expression. (D) Western blot showing reduced expression of PU.1, TLR2, and TLR4 with TRP120 stimulation. Representative data are shown (*n* = 4).

We determined that *E. chaffeensis* activates the canonical Notch signaling pathway, and this pathway suppresses TLR2/4 expression during infection. Since TRP120 plays a critical role in activation of Notch components and target gene expression, we sought to evaluate their effect on PU.1 inhibition and TLR2/4 expression. To test this, THP-1 cells were treated with 1 µg/ml of either TRP120 or thioredoxin (control) in soluble form, or latex beads were coated with TRP120 and incubated at 37°C for 24 h. TRP120-treated and control cells were then stimulated with LPS (100 ng/ml) for 1 h, and expression of PU.1, TLR2, and TLR4 was determined using immunofluorescence microscopy. Figure 9A shows strong PU.1 expression in the nucleus of thioredoxin-treated cells in response to LPS stimulation. However, after LPS treatment, reduction in the expression level of PU.1 in response to TRP120 stimulation (both bead bound and in suspension) was observed. Since PU.1 transcription factor regulates TLR2/4 expression, we also determined the effect of TRP120 in TLR expression using the same method. Thioredoxin-treated cells showed strong TLR2 expression in both cytoplasm and nucleus of the cells in response to LPS; however, cells stimulated with TRP120 showed reduced expression of TLR2 (Fig. 9B). Similar results were found when TLR4 expression was measured in response to TRP120 stimulation (Fig. 9C). Western blotting was done to analyze the protein expression of PU.1, TLR2, and TLR4 in control and TRP120-stimulated (bead-bound and soluble) THP-1 cells in response to LPS. Decreased expression of all three proteins was observed, which further validated our IFA data (Fig. 9D). These studies demonstrate that TRP120 effector protein plays a direct role in modulating TLR expression during *E. chaffeensis* infection.

## DISCUSSION

In recent years, the essential role of Notch signaling in determining the fate of T cells, B cells, dendritic cells (DCs), monocytes, and macrophages at different stages of life has been widely studied (24, 41–45). In this study, we demonstrated that *E. chaffeensis* and more specifically the TRP120 effector activates Notch signaling and exploits this evolutionarily conserved pathway for its survival. In addition, we have gained an understanding of the mechanisms behind *E. chaffeensis*-mediated activation of Notch signaling and how it affects PRR expression and ehrlichial survival in monocytes.

*E. chaffeensis* successfully evades innate immune recognition and natural phagocytic killing, but the mechanisms are unknown. However, the TRP effectors play important roles in immune subversion by chromatin manipulation or by directly interacting with host proteins to modulate cellular processes. More recently, we demonstrated that TRP effectors cause activation of the Wnt signaling pathway, which facilitates host cell entry by inducing phagocytosis and promotes intracellular survival (10). Previously, we reported that TRP120 interacts with ADAM17 metalloprotease and binds to the promoter region of *notch1* (9, 11). Since Notch is a critical signaling pathway in monocytes and macrophages (24, 44, 46–48), we hypothesized that *E. chaffeensis* TRP120 directly activates the Notch signaling to promote survival. During *E. chaffeensis* infection, nuclear localization of transcriptionally active NICD and induction of a panel of Notch genes expressed in monocytes were observed. We demonstrated comprehensive molecular modulation of Notch signaling pathway genes and downstream target genes at different times after infection. This suggests that activation of the canonical Notch signaling pathway occurs at both early and late phases of ehrlichial infection. Induction of TRP120-interacting protein ADAM17 was also observed during ehrlichial infection. ADAM17, also known as tumor necrosis factor-converting enzyme, or TACE, is an important regulator of Notch signaling and plays a critical role in regulation of different cellular events, including proliferation and migration. Thus, ADAM17 is implicated in different human diseases, including cancer, and is a promising target for treatment (49). Several studies reported induction of ADAM17 and its anti-inflammatory effect during bacterial infection (50). Both *Staphylococcus aureus* and *Pseudomonas aeruginosa* induce ADAM17 in airway epithelial cells and regulate inflammation by neutralizing interleukin-6 (IL-6) (51) and tumor necrosis factor alpha (TNF-α) (52). Based on our previous study showing direct TRP120-ADAM17 interaction and the colocalization studies herein, we propose that this interaction could activate ADAM17, resulting in Notch1 cleavage and pathway activation. It is not clear if TRP120 interacts directly with Notch1, but it is possible that such an interaction occurs. Moreover, TRP120-induced nuclear translocation of NICD and induction of genes associated with Notch signaling demonstrate that this effector protein plays important role in canonical Notch pathway activation. Compared to the bacterial infection, the TRP120, especially the soluble form, showed a stronger effect on modulation of Notch pathway gene expression. It might be because of the higher concentration of purified TRP120 used in the experiments and also because the soluble protein has more surface area for interaction with the receptor complex than the bead-bound protein. This study revealed a novel mechanism whereby *E. chaffeensis* uses TRP120 to activate the Notch signaling pathway by interacting with and inducing Notch receptor complex components.

Notch plays critical role in regulating the maturation and function of different immune cells, including monocytes and macrophages. Therefore, Notch signaling-mediated changes in the properties of these cells may also affect bacterial growth. Pharmacological inhibitors against Notch transcription factor protein (SAHM1), γ-secretase enzyme (DAPT), and siRNAs against Notch1, ADAM17, and RBPjκ confirmed that the canonical Notch signaling pathway is required for ehrlichial survival. Differences in ehrlichial loads observed between different siRNA treatments (at day 1 p.i.) might occur because the role of these targets varies at different stages of infection. Also, the siRNAs might vary in their efficacy, and it is possible that the ADAM17 and RBPjκ siRNAs required more time for efficient knockdown. Together, the gene expression studies correlated strongly with inhibitor data supporting the conclusion that canonical Notch signaling is required for ehrlichial survival and may be a novel target for developing therapeutics.

TLRs, the key PRR expressed on monocytes and macrophages, play critical roles in host defense against any invading pathogens, including *E. chaffeensis* (25, 31, 53). Interaction of TLR with PAMPs causes activation of a signaling transduction cascade and elicits a proinflammatory cytokine and chemokine response. This profound innate immune response helps in the elimination of invading organisms (29, 54, 55). *E. chaffeensis* lacks the genes required for biosynthesis of LPS and peptidoglycan, which presumably prevents detection by PRRs (56). However, this unusual cell wall structure does not protect them fully from recognition by immune cells. Using TLR4^−/−^ mice, it has been shown that inhibition of TLR4 causes decreased nitric oxide and IL-6 secretion by macrophages, which results in short-term persistence of ehrlichial infection (25). Recently, another study reported that absence of TLR2 impaired ehrlichial elimination and the TLR2-dependent immune response was protective against the infection (31). *E. chaffeensis* causes decreased expression of TLR2/4 in monocytes, which involves inhibition of ERK1/2, p38 MAPK, and PU.1 activity (2). However, the specific mechanism that *E. chaffeensis* utilizes to downregulate TLR expression and the upstream signaling molecule that inhibits the ERK1/2 and p38 MAPK pathways were unknown.

Multiple studies have reported cross talk between TLR and Notch signaling pathways (44, 47). TLRs have been shown to activate Notch signaling through a JNK-dependent pathway to regulate inflammatory response (57). However, TLRs can also cause suppression of Notch signaling in macrophages (58). Combined effects of Notch and TLR signaling in the induction of the *hes* and *hey* genes have been shown to function as part of a feedback loop and attenuate the production of cytokines (e.g., IL-6 and IL-12). Association of Notch signaling in modulation of ERK1/2 and regulation of TLR4-triggered cytokine production has recently been reported (24). However, to our knowledge, no previous studies have reported Notch-mediated regulation of TLR response during bacterial infection. Herein, we demonstrated that *E. chaffeensis* causes inhibition of PU.1 and TLR2 and -4 expression by directly activating the Notch pathway. The underlying mechanism involves Notch-mediated inhibition of ERK1/2 and p38 MAPK activation. Based on our observation and previous findings, we also expected less NF-κB (p65/50) activation by LPS stimulation in *E. chaffeensis*-infected cells (34). Although Notch PCR array data showed induction of NF-κB1 (p105) and NF-κB2 (p100) genes in THP-1 cells infected with *E. chaffeensis* or stimulated with TRP120, this finding does not contradict the previous findings, which reported inhibition of NF-κB during infection. NF-κB1 and NF-κB2 proteins serve as both NF-κB precursors and inhibitors. The classical inhibitors IκBα, -β, -δ, and -ε sequester the transcription factor NF-κB in the cytoplasm by masking their nuclear localization signal (NLS) (59). Unlike these inhibitors p105 and p100 proteins assemble into high-molecular-weight complexes and bind NF-κB subunits (60). Previous studies reported *M. bovis* upregulation of Notch1 expression and activation of Notch signaling leading to activation of SOCS3 (suppression of cytokine signaling 3), which is a negative regulator of signaling of multiple cytokines and TLR (26, 61). Herein, we provide data to support a different mechanism, whereby *E. chaffeensis* T1S effector protein TRP120 can manipulate Notch signaling to regulate immune recognition through inhibition of PRR expression to promote survival. Although we demonstrated that TLR2 and TLR4 expression is regulated during *E. chaffeensis* infection, it is possible that other TLRs can also be regulated by the Notch signaling during infection. TRP120 is found on the surface of the infectious dense-core form, but is also secreted into the host cell, where it interacts with a variety of host cell targets and DNA (9, 11, 62). TRPs can also be released from the *E. chaffeensis*-infected cell during infection (63). Data presented in this study demonstrated that TRP120 bound to a substrate or in soluble form can activate Notch signaling pathway and modulate PU.1 and TLR2/4 expression. These findings are significant as they suggest that the effects of *E. chaffeensis* TRP120 on Notch pathway activation not only occur through direct bacterium-host interactions but may have systemic effects through the release of soluble TRP120 that could interact with uninfected cells to downregulate innate immunity and promote infection.

In addition to TLR-mediated innate immune responses, another important mechanism involved in the elimination of intracellular bacteria is autophagy, which appears to be inhibited during ehrlichial infection (64). Studies indicate that Notch and Wnt signaling plays a crucial role in inhibition of autophagy through activation of the mTOR pathway and regulation of autophagy receptor p62 expression (65–68), and preliminary data (T. T. Lina, and J. W. McBride, unpublished data) from our laboratory suggest Wnt and Notch signaling is involved in inhibition of autophagy during ehrlichial infection. Hence, *Ehrlichia* inhibition of TLR recognition is likely not the only innate immune mechanism that is affected by Notch signaling. Further studies are needed to fully understand the cross talk between the Wnt and Notch signaling pathways and how these pathways act synergistically to inhibit host innate immune responses such as autophagy to promote ehrlichial survival.

**FIG 10:**
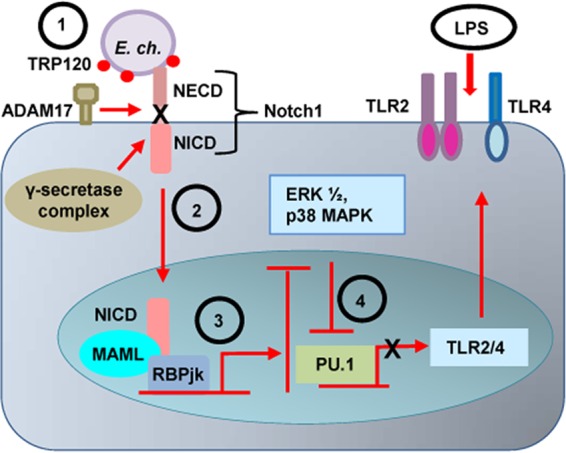
Proposed model for *E. chaffeensis* TRP120-mediated activation of canonical Notch signaling pathway and inhibition of TLR2/4 expression. (Step 1) *E. chaffeensis* TRP120 effector interaction with ADAM17 activates the metalloprotease, resulting in cleavage of the substrate Notch1, and subsequent cleavage by γ-secretase causes (step 2) nuclear translocation of NICD, the transcriptionally active form of which binds with RBPjκ and MAML proteins. This triprotein complex activates transcription of Notch target genes, which causes inhibition of the ERK1/2 and p38 MAPK pathways (step 3). The downstream transcription factor PU.1 expression is repressed, which causes further inhibition of monocyte TLR2/4 expression (step 4). Inhibition of TLR2/4 expression causes both inhibition of *E. chaffeensis* recognition and TLR-mediated proinflammatory cytokine production needed for the activation of monocytes and clearance of ehrlichiae.

Our limited understanding of the molecular pathogen-host interactions and cellular pathways usurped by *Ehrlichia* as well as those of other obligate intracellular microbes is a major impediment to defining the mechanisms that enable ehrlichial intracellular survival and development of next-generation therapeutics aimed at mechanistically defined targets. This study reveals a novel effector-dependent mechanism, which involves interaction with the ADAM17 and Notch1 and activation of canonical Notch signaling pathway in monocytes, a primary target of *E. chaffeensis*, to modulate ERK1/2 and p38 MAPK pathways and regulate TLR2/4 expression (Fig. 10). Hence, this study demonstrates the importance of Notch pathway in ehrlichial survival and provides a new target for the development of a novel therapeutic approach against ehrlichial infection that may be applicable to other intracellular pathogens, in which exploitation of such conserved cellular pathways is required for pathogen survival.

## MATERIALS AND METHODS

### Cell culture and cultivation of *E. chaffeensis*.

Human monocytic leukemia cells (THP-1) were propagated in RPMI 1640 with l-glutamine and 25 mM HEPES buffer (Invitrogen), supplemented with 1 mM sodium pyruvate (Sigma, St. Louis, MO), 2.5 g/liter d-(+)-glucose (Sigma), and 10% fetal bovine serum at 37°C in a 5% CO_2_ atmosphere. *E. chaffeensis* (Arkansas strain) was cultivated in THP-1 cells as previously described (8).

### Antibodies and inhibitors.

The polyclonal mouse anti-TRP120 antibody used in this study was previously described (69). A convalescent-phase anti-*E. chaffeensis* dog serum, which was derived from an experimentally infected dog, was described previously (40). Other antibodies that were used in this study include anti-Hes1 (ab71559) (Abcam, Cambridge, MA), anti-Notch1 (C-20), anti-cleaved Notch1 (m1711), anti-α-tubulin (B7), anti-TLR2 (TL2.1), anti-TLR4 (15), anti-PU.1 (A-7) (Santa Cruz Biotechnology, NY), anti-ADAM17 (anti-TACE) (D22H4), anti-Notch1 (D1E11), anti-RBPSUH (D10A4) (Cell Signaling Technology, Inc.), and anti-GAPDH (clone 6C5, EMD) (Millipore, CA). For inhibition of the Notch signaling pathway, the following inhibitors were used: γ-secretase inhibitor IX, named DAPT {*N*-[*N*-(3, 5-difluorophenacetyl-l-alanyl)]-*S*-phenylglycine *t*-butyl ester} (Calbiochem, Canada), and Notch transcription factor inhibitor SAHM1 (Calbiochem, Canada).

### siRNAs and transfection.

To knockdown Notch signaling components, THP-1 cells (1 × 10^5^/well on a 96-well plate) were transfected with siRNA for ADAM17 (TACE), RBP-jκ, or Notch1 (Santa Cruz Biotechnology, NY) using the Lipofectamine 2000 reagent (Life Technologies, CA) according to the manufacturer’s instructions with a cocktail of 5 pmol siRNA or a negative-control siRNA (Santa Cruz Biotechnology, NY).

### RT-PCR.

Total RNA from *E. chaffeensis*-infected, TRP120- or thioredoxin-stimulated and control THP-1 cells was isolated using an RNeasy minikit (Qiagen) according to the manufacturer’s instructions. On-column DNA digestion was performed using the RNase-free DNase set (Qiagen). cDNA was synthesized from 1 µg of total RNA using a qScript cDNA SuperMix kit (Quanta Biosciences). The gene expression level of the target host genes was quantitated by qPCR using Brilliant II SYBR green qPCR master mix (Agilent Technologies) with gene-specific primers and a thermal cycling protocol consisting of an initial denaturation step of 95°C for 10 min and 40 cycles of 95°C for 30 s, 58°C for 1 min, and 72°C for 30 s. Gene expression values were calculated on the basis of the threshold cycle (2^−ΔΔ*CT*^) method and normalized with GAPDH.

### Human Notch signaling pathway PCR array.

The human Notch signaling pathway RT^2^ Profiler PCR array (Qiagen) was used according to the manufacturer’s protocol to determine the expression of 84 genes, which include genes coding for receptors, and ligands, as well as receptor processing, and transcription factor genes associated with Notch signaling and putative Notch target genes. Briefly, RNA was collected from *E. chaffeensis*-infected and uninfected cells, cells stimulated with TRP120- or thioredoxin-coated FluoroSpheres (sulfate microsphere beads) or TRP120 alone, using the RNeasy minikit (Qiagen). RNA purification, genomic DNA elimination, cDNA synthesis, and the PCR array were performed as previously described (10).

### Immunofluorescence microscopy.

Uninfected or *E. chaffeensis*-infected cells or THP-1 cells stimulated with TRP120- or /thioredoxin-coated beads and TRP120 in soluble form were cytospun onto glass slides, fixed for 15 min using 3% paraformaldehyde in phosphate-buffered saline (PBS), blocked and permeabilized for 30 min using 0.3% Triton X-100 and 2% bovine serum albumin (BSA) in PBS at room temperature. Cells were then incubated with primary antibodies rabbit anti-TRP120 (1:1,000), dog anti-*E. chaffeensis* serum (1:100), goat anti-NICD (1:50), mouse anti-ADAM17 (1:50), goat anti-Notch1 (1:50), rabbit anti-Hes1 (1:50), mouse anti-TLR2 (1:50), mouse anti-TLR4 (1:50), and mouse anti-PU.1 (1:50) for 1 h, washed, and incubated with Alexa Fluor 488 IgG (H+L) and Alexa Fluor 568 IgG (H+L) secondary antibodies (1:100 [Molecular Probes]) for 30 min. Slides were mounted with ProLong Gold antifade reagent with DAPI (4′,6-diamidino-2-phenylindole) (Invitrogen) after washing. HeLa cells transfected with the GFP-TRP120 plasmids or GFP control plasmids were fixed in chamber slides, permeabilized, and stained with anti-ADAM17 using the same protocol. Images were obtained using an Olympus BX61 epifluorescence microscope and analyzed using Slidebook software (version 5.0; Intelligent Imaging Innovations, Denver, CO).

### Pharmacological inhibitor treatment and determination of bacterial load.

THP-1 cells were treated with DAPT, SAHM1, or dimethyl sulfoxide (DMSO), incubated for at least 1 h, and then infected with cell-free *E. chaffeensis* at a multiplicity of infection (MOI) of 50. According to the 50% inhibitory concentrations (IC_50_s) and previously published concentrations, 5 µg/ml of DAPT or 10 µM SAHM1 was used to treat the cells unless otherwise stated (38, 70). At days 1 and 2 p.i., cells were collected, and infection was determined by either calculating the percentage of infected cells after Diff-Quick staining or by determining the *dsb* copy number using qPCR as previously described (71). Infected culture without the inhibitors and uninfected cells were used as positive and negative controls, respectively. The absolute *E. chaffeensis* dsb copy number was determined using a standard curve and was normalized to qPCR-detected levels of the host genomic *gapdh* gene. To confirm that host cell death did not account for decreased ehrlichial inclusions, differences in cell viability were assessed at days 1, 2, and 3 p.i. using trypan blue staining.

### Western immunoblot.

THP-1 cells infected with *E. chaffeensis* in the presence of DMSO or Notch inhibitor SAHM1 and uninfected cells were harvested after 1 and 2 days p.i. and LPS stimulation (100 ng/ml for 1 h). Cell lysates were prepared as previously described (3). Approximately 20 µg of total proteins was separated by sodium dodecyl sulfate-polyacrylamide gel electrophoresis (SDS-PAGE) and transferred to nitrocellulose membrane using a semidry transfer apparatus. Mouse anti-ADAM17, rabbit anti-Hes1, mouse anti-α-tubulin, mouse anti-PU.1, mouse anti-TLR4, mouse anti-TLR2, and mouse anti-GAPDH were used. For the detection, horseradish peroxidase-labeled goat anti-rabbit or mouse IgG (heavy and light chains) conjugate (Kirkegaard & Perry Laboratories, Gaithersburg, MD) was used. SuperSignal West Dura chemiluminescent substrate (Thermo Scientific) was used for detection of Hes1 protein, and ECL enhanced chemiluminescent Western immunoblot substrate (Thermo Scientific) was used for others.

### Bead assay.

*E. chaffeensis* TRP120 protein (thioredoxin fused) was expressed and purified as described previously (6, 63, 69). Purified proteins were desalted (Zeba Spin desalting column; Thermo Scientific) to change the buffer to 40 mM MES [2-(*N*-morpholino)ethanesulfonic acid]. FluoroSpheres (sulfate microsphere beads; 1.0 µm, yellow-green fluorescent [Invitrogen]) were coated with recombinant purified TRP120 or thioredoxin using the following protocol. Briefly, 10 µl of beads (~3.6 × 10^8^ beads) was washed two times with 10 volumes of 40 mM MES buffer (5,000 × *g* for 5 min), resuspended in 10 µg of TRP120 desalted protein in 500 µl MES buffer, and incubated at room temperature for 2 h in a rotor. After incubation, beads were washed twice with 500 µl MES buffer (10,000 × *g* for 8 min) and resuspended in RPMI medium. Since these beads are light sensitive, they were also protected from exposure to light. TRP120- or thioredoxin-coated beads were used to treat THP-1 cells for different time points, and the cells were incubated at 37°C with 5% CO_2_. After incubation, unbound beads were washed by centrifugation at least 4 times at 400 × *g*.

### Bio-Plex.

The levels of total and phosphorylated ERK1/2 and p38 MAPK proteins in THP-1 cells infected with *E. chaffeensis* in the presence and absence of the Notch inhibitor SAHM1 (10 µM) and with or without LPS stimulation (100 ng/ml) were measured using the Luminex array (Millipore, Billerica, MA) according to manufacturer’s instructions. Samples were analyzed using Bio-Plex Manager software (Bio-Rad).

### Statistics.

The results are expressed as the means ± standard deviation (SD) of data obtained from at least three independent experiments done with triplicate sets per experiment, unless otherwise indicated. Differences between means were evaluated by using two-tailed Student’s *t* test. *P* values of <0.05 were considered statistically significant.

## SUPPLEMENTAL MATERIAL

Figure S1  List of 84 genes analyzed in the Notch PCR array. Download Figure S1, TIF file, 0.2 MB

Figure S2  Dose-dependent inhibition of *E. chaffeensis* after treatment with Notch inhibitors. THP-1 cells were treated with (A and B) DAPT and (C and D) SAHM1. Cells were infected with *E. chaffeensis* after 1 h posttreatment. Ehrlichial loads were determined at 24 and 48 h p.i. by measuring the percentage of infected cells by counting 100 Diff-Quik-stained cells. Download Figure S2, TIF file, 0.1 MB

Figure S3  Expression array analysis of Notch signaling genes after stimulation with TRP120 (in suspension). (A) Heat map showing relative expression levels of Notch signaling genes after TRP120 (soluble) stimulation. The scale bar shows color-coded differential expression from the mean gene expression level of thioredoxin-stimulated cells. The degree of color represents the level of induction (red)/repression (green). (B) Scatter plot showing the Notch gene expression after TRP120 (soluble) stimulation. A red dot represents increased gene expression, a black dot represents no significant change of expression, and a green dot represents decreased gene expression compared to that of control cells. The cutoff was 2-fold. Download Figure S3, TIF file, 0.3 MB
