# Supplementary figures and images for: Ehrlichia chaffeensis TRP120 Activates Canonical Notch Signaling To Downregulate TLR2/4 Expression and Promote Intracellular Survival
Source: mBio. 2016 Jul 5;7(4):e00672-16. doi: 10.1128/mBio.00672-16 (PMC4958247; doi:10.1128/mBio.00672-16)

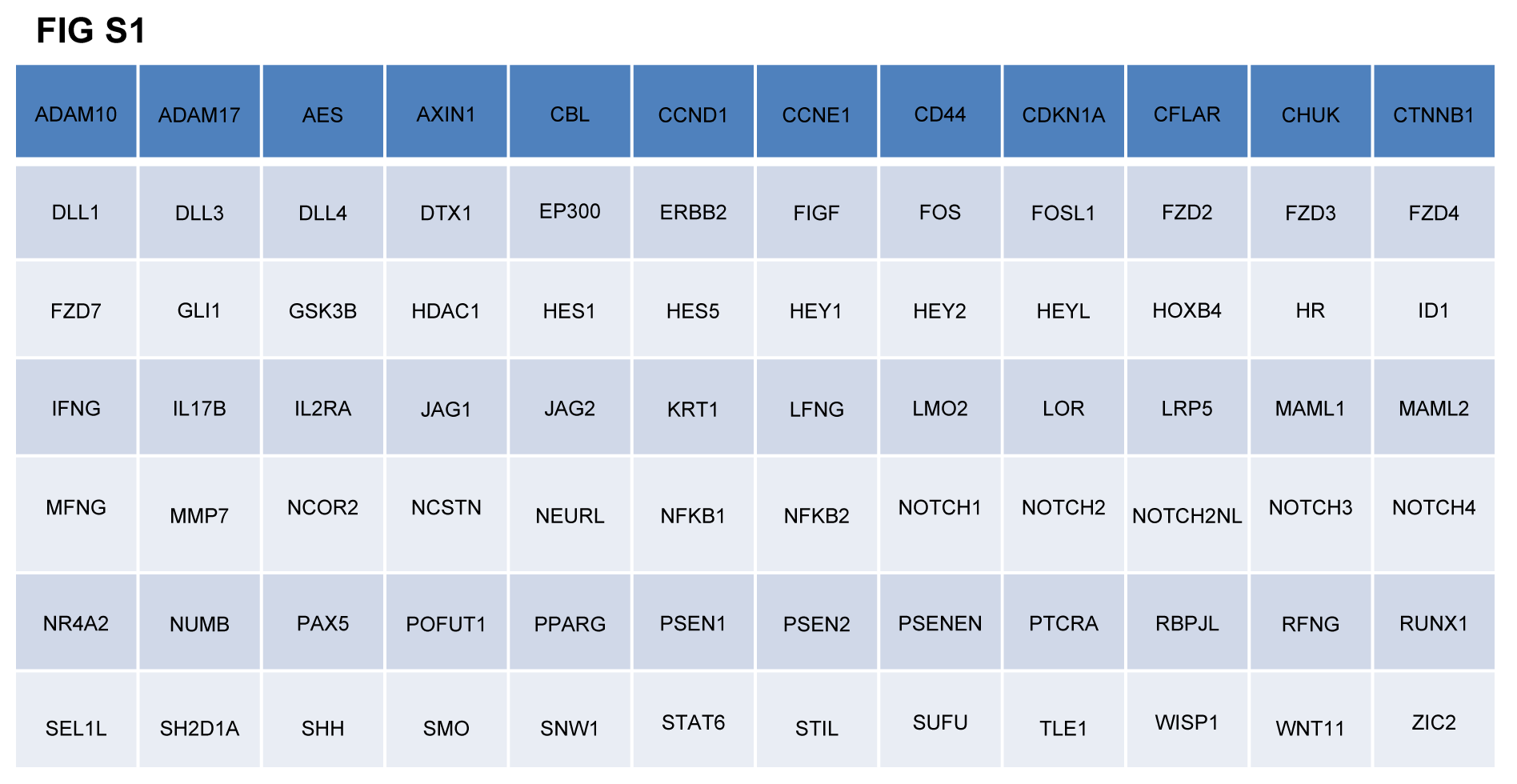

Supplement: Figure S1 — List of 84 genes analyzed in the Notch PCR array. Download [file mbo003162884sf1.tif]

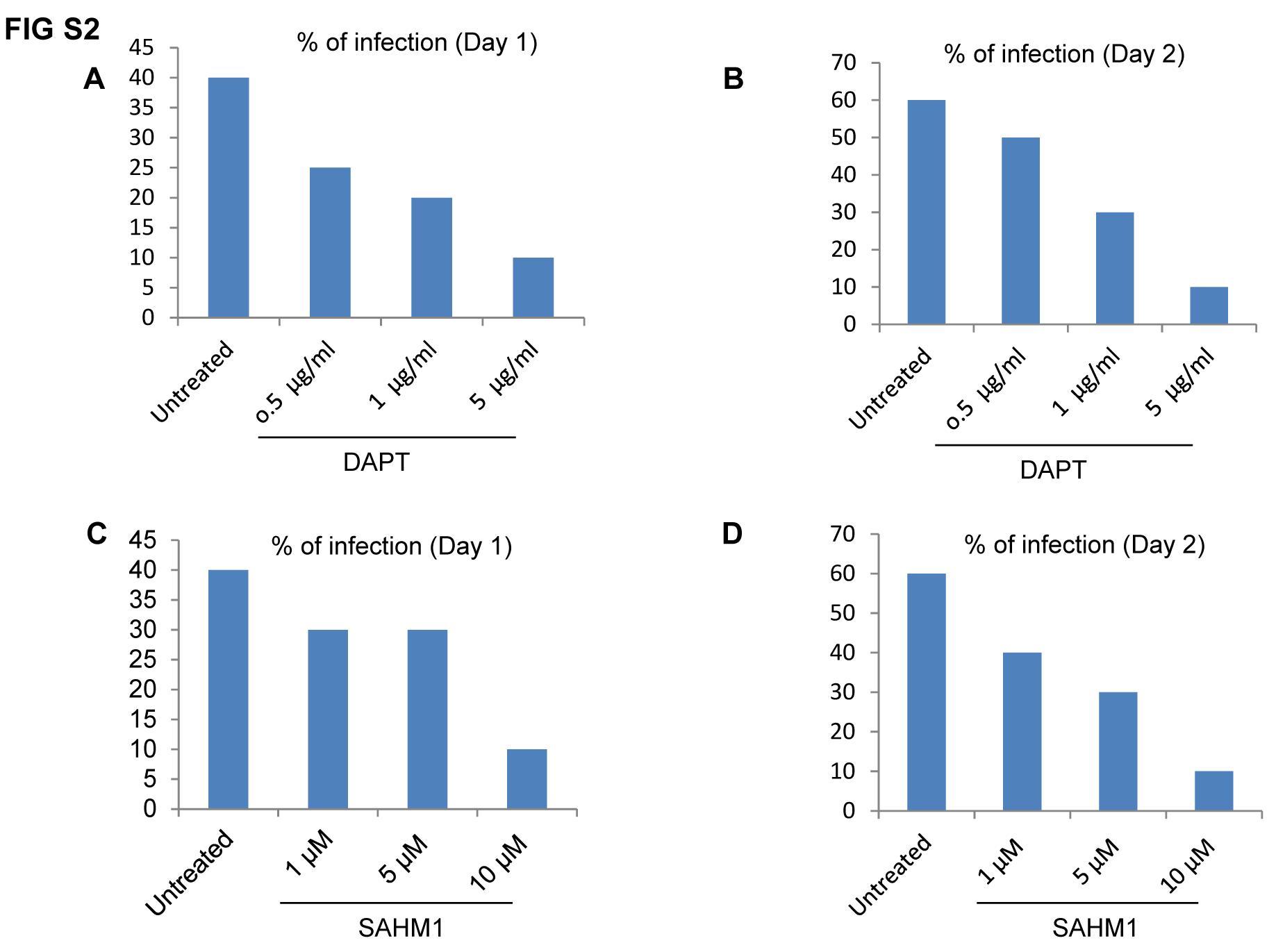

Supplement: Figure S2 — Dose-dependent inhibition of E. chaffeensis after treatment with Notch inhibitors. THP-1 cells were treated with (A and B) DAPT and (C and D) SAHM1. Cells were infected with E. chaffeensis after 1 h posttreatment. Ehrlichial loads were determined at 24 and 48 h p.i. by measuring the percentage of infected cells by counting 100 Diff-Quik-stained cells. Download [file mbo003162884sf2.tif]

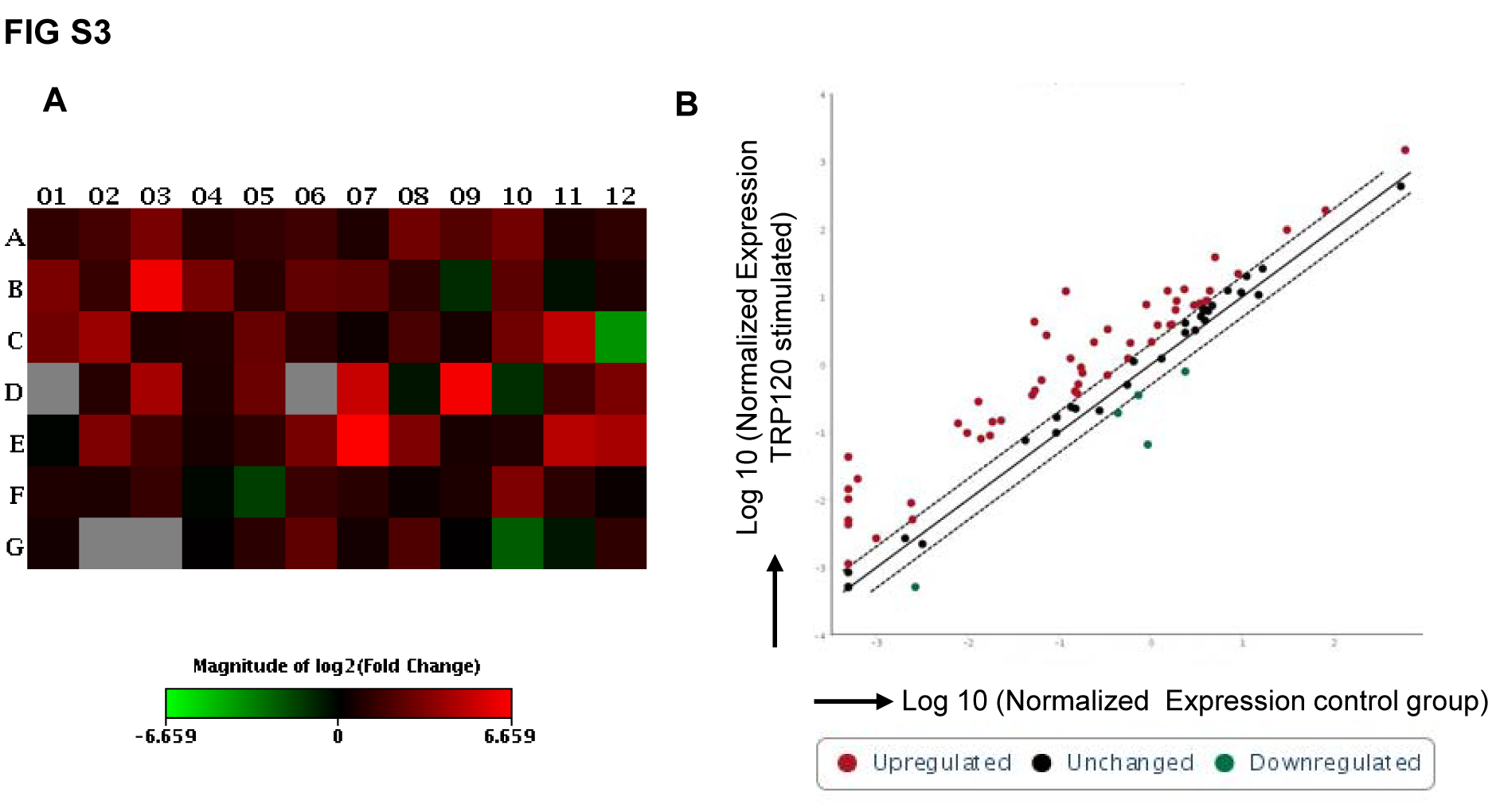

Supplement: Figure S3 — Expression array analysis of Notch signaling genes after stimulation with TRP120 (in suspension). (A) Heat map showing relative expression levels of Notch signaling genes after TRP120 (soluble) stimulation. The scale bar shows color-coded differential expression from the mean gene expression level of thioredoxin-stimulated cells. The degree of color represents the level of induction (red)/repression (green). (B) Scatter plot showing the Notch gene expression after TRP120 (soluble) stimulation. A red dot represents increased gene expression, a black dot represents no significant change of expression, and a green dot represents decreased gene expression compared to that of control cells. The cutoff was 2-fold. Download [file mbo003162884sf3.tif]
